# The role of DNA methylation in the pathogenesis of type 2 diabetes mellitus

**DOI:** 10.1186/s13148-020-00896-4

**Published:** 2020-07-11

**Authors:** Sanabil Ali Hassan Ahmed, Suraiya Anjum Ansari, Eric P. K. Mensah-Brown, Bright Starling Emerald

**Affiliations:** 1 Department of Anatomy, College of Medicine and Health Sciences, United Arab Emirates University, PO Box 17666, Al Ain, Abu Dhabi, United Arab Emirates; 2 Department of Biochemistry, College of Medicine and Health Sciences, United Arab Emirates University, PO Box 17666, Al Ain, Abu Dhabi, United Arab Emirates

**Keywords:** Diabetes mellitus, DNA methylation, Type 2 diabetes mellitus, Hypermethylation, Hypomethylation

## Abstract

Diabetes mellitus (DM) is a chronic condition characterised by β cell dysfunction and persistent hyperglycaemia. The disorder can be due to the absence of adequate pancreatic insulin production or a weak cellular response to insulin signalling. Among the three types of DM, namely, type 1 DM (T1DM), type 2 DM (T2DM), and gestational DM (GDM); T2DM accounts for almost 90% of diabetes cases worldwide.

Epigenetic traits are stably heritable phenotypes that result from certain changes that affect gene function without altering the gene sequence. While epigenetic traits are considered reversible modifications, they can be inherited mitotically and meiotically. In addition, epigenetic traits can randomly arise in response to environmental factors or certain genetic mutations or lesions, such as those affecting the enzymes that catalyse the epigenetic modification. In this review, we focus on the role of DNA methylation, a type of epigenetic modification, in the pathogenesis of T2DM.

## Introduction

### Diabetes mellitus

Diabetes mellitus (DM) is a chronic condition characterised by persistently elevated glucose levels in the bloodstream (i.e., hyperglycaemia) [1] and pancreatic β cell dysfunction [2]. In general, the disease is classified into three types, namely, type 1 DM (T1DM), type 2 DM (T2DM), and gestational DM (GDM), with T2DM accounting for approximately 90% of diabetes cases worldwide. In T2DM, the associated hyperglycaemia can be due to inadequate pancreatic insulin production or a weak cellular response to insulin signalling (i.e., insulin resistance). In addition, the development of T2DM depends, in part, on the balance between pancreatic β cell proliferation and apoptosis [1, 3].

T2DM is a multifactorial disease, with its aetiology affected by multiple genes (i.e., polygenic) in addition to environmental factors. Indeed, genome-wide association studies (GWASs) have linked aberrations in more than 40 different genes with an increased risk of T2DM. These genes are involved in the regulation of various biological processes, including cellular development, differentiation, and physiological functions. Environmental factors that have been associated with an increased risk of T2DM include age, obesity, and lack of physical activity [2].

The persistent hyperglycaemia in DM can ultimately lead to adverse complications, such as neuropathy, retinopathy, nephropathy, and cardiovascular diseases (CVDs). As a compounding factor, there is a prolonged pre-detection period in T2DM, during which one third to one half of all patients may go undiagnosed due to a lack of clinical symptoms. Indeed, some patients are only diagnosed with T2DM after the manifestation of complications associated with T2DM-induced hyperglycaemia [1, 3].

**Fig. 1:**
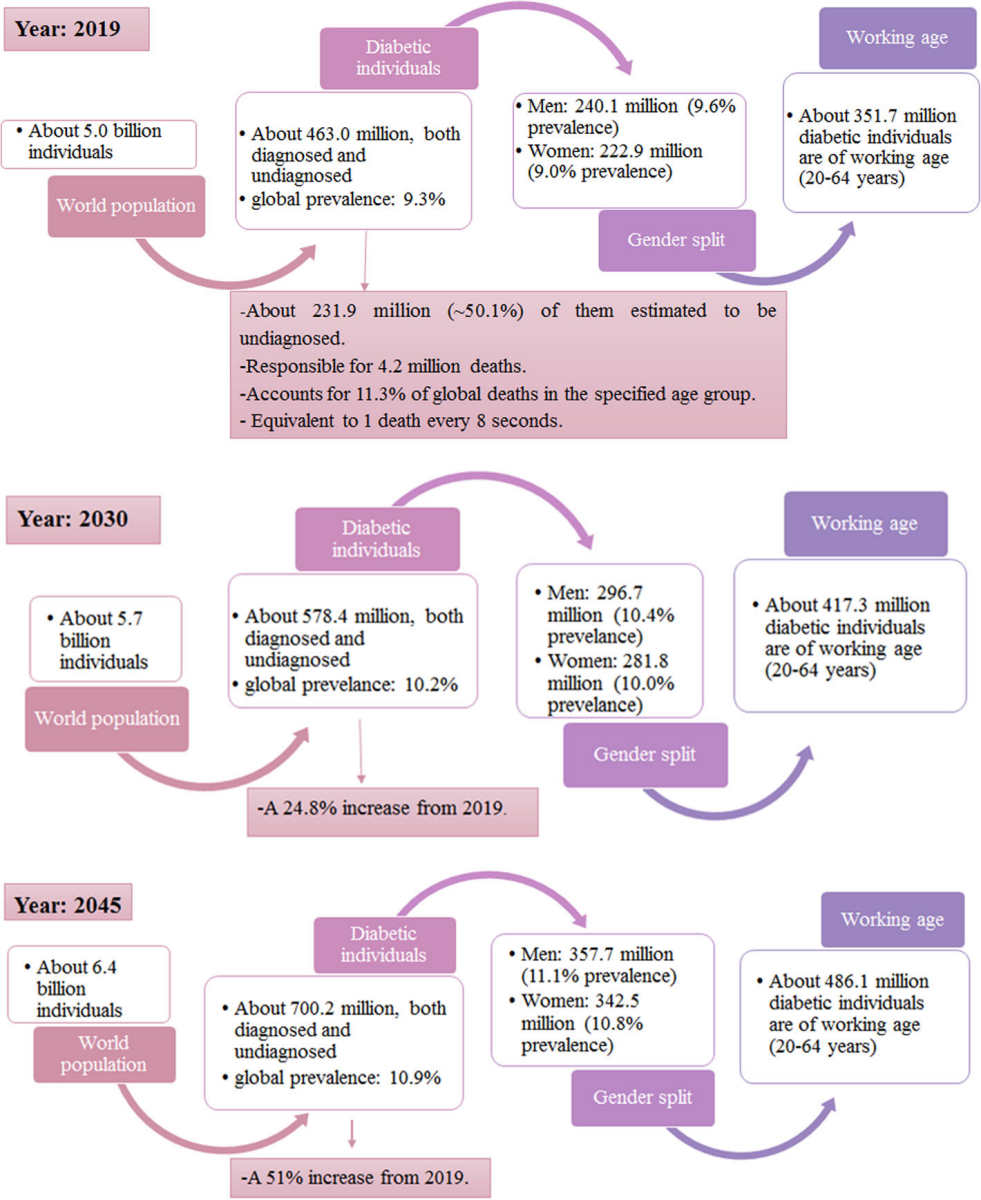
Worldwide DM statistics in 2019 for the age group 20–79 years old and the projections for years 2030 and 2045

The latest statistics of the prevalence of DM (as of 2019) and the predicted statistics of the prevalence of DM for the years 2030 and 2045 are summarised in Fig. 1 [3]. Despite the high prevalence of DM, the exact mechanisms underlying the development of GDM, T1DM, and T2DM are not fully understood [1, 3]. Therefore, a greater understanding of the aetiology of DM is necessary to develop improved prevention and diagnostic tools and treatments for associated complications.

### Epigenetics

An epigenetic trait is a stably heritable phenotype resulting from certain changes in gene expression that do not alter the underlying DNA sequence [4]. Epigenetic traits are considered reversible modifications that can be inherited mitotically and meiotically. However, they can also randomly arise due to various environmental factors or genetic mutations such as those affecting the enzymes that catalyse the epigenetic modification [5, 6]. Important epigenetic modifications include DNA methylation; posttranslational histone tail modifications, such as methylation, phosphorylation, acetylation, and regulatory RNAs [7].

In general, epigenetic modifications do not occur independently but rather crosstalk with and regulate one another to create an epigenetic profile, which changes the function of the genome (gene expression) by altering the chromatin structure. The particular epigenetic profile can either lead to gene activation by relaxing the chromatin structure, thus allowing the transcription machinery to access the DNA, or to gene silencing by tightly packaging the chromatin structure and inhibiting access to the DNA [6]. Accordingly, the epigenetic profile has been shown to modulate gene expression in different cell types, developmental stages, and in health and disease states [8].

**Fig. 2:**
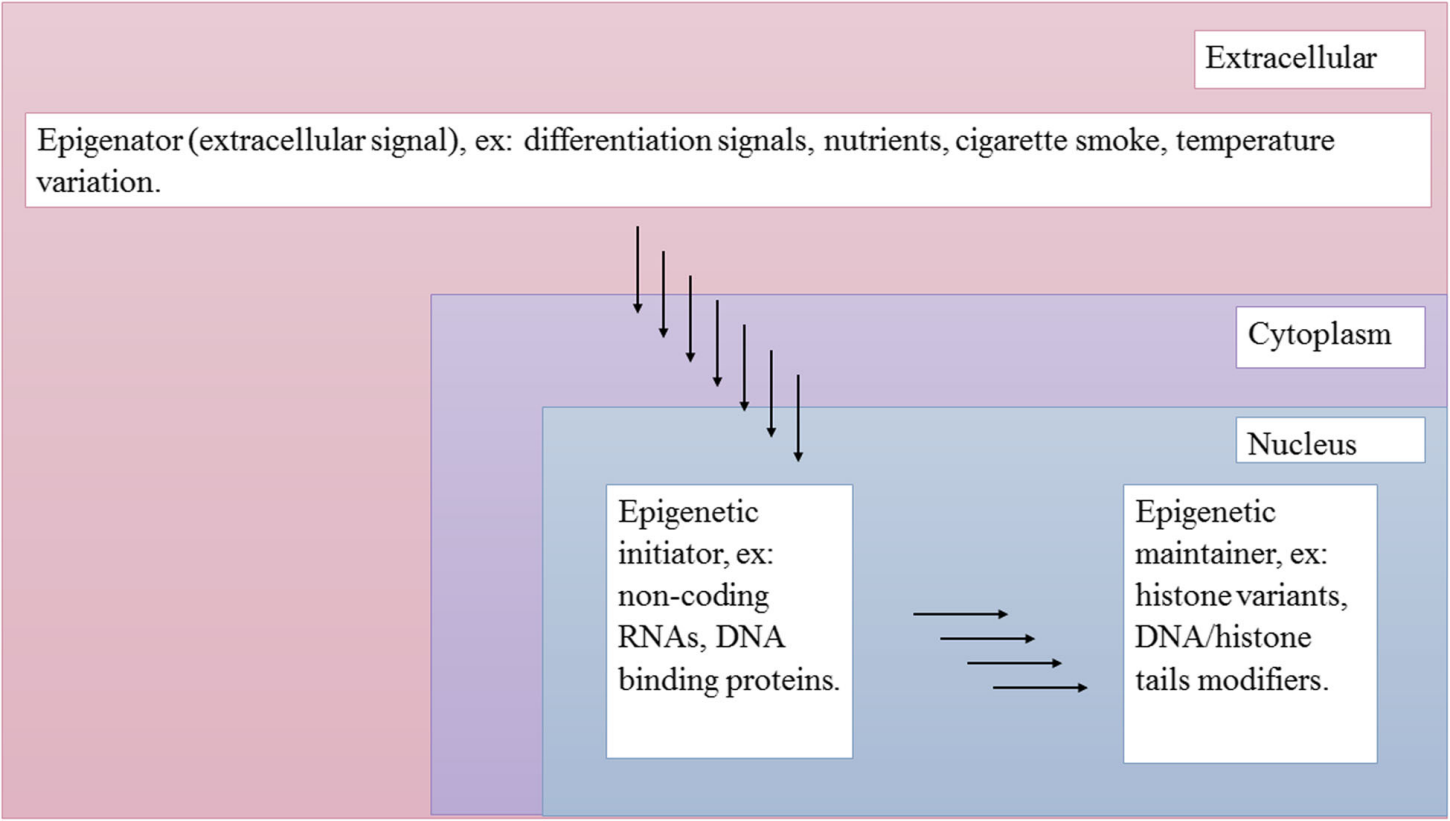
The epigenetic pathway and the three categories of signals proposed. The extracellular epigenator signal triggers the initiation of the epigenetic pathway. The epigenetic initiator receives the signal from the epigenator and determines the chromatin state for establishing the epigenetic pathway. The epigenetic maintainer sustains the chromatin state in succeeding generations

Three categories of signals that stimulate an epigenetic state have been proposed, namely, epigenators, epigenetic initiators, and epigenetic maintainers (Fig. 2). The epigenator signal is environmentally induced and triggers an intracellular pathway. It can be a modification-based event or a protein–protein interaction that releases the dormant activity of the epigenetic initiator. An example of an epigenator signal is temperature (in plants). The extracellular epigenator signal is received and transmitted by the intracellular epigenetic initiator signal, which translates the signal by initiating events required for establishing the signalled chromatin state at the chromatin location. The initiator signal can be non-coding RNAs, DNA-binding proteins and/or any entity capable of recognising DNA sequences and defining the chromatin state to be generated. An epigenetic maintainer signal maintains the chromatin state over subsequent generations, although not sufficiently enough to initiate it. This signal maintainer preserves the epigenetic profile in terminally differentiated cells and throughout the cell cycle. Examples include the DNA methylation of CpG islands [4].

### DNA methylation

DNA methylation, which naturally occurs as a result of DNA replication, is the covalent addition of a methyl group (–CH_3_) to the 5’ position of the cytosine residue in the dinucleotide 5’-cytosine-phosphodiester bond-guanine-3’ (5’-CpG-3’) to form 5-methylcytosine (5mC). CpG DNA methylation is reversible [6, 9]. CpG islands have a high CpG density (> 50%) and are primarily located in the promoter region of genes but can also be found in enhancer and intragenic regions [5, 6]. Although 70% of CpG dinucleotides are methylated in humans, CpG dinucleotides of germ-line cells and promoters of somatic cells are relatively unmethylated [9].

In general, promoter CpG island hypomethylation is associated with transcriptional activation, whereas the hypermethylation of CpG islands is associated with transcriptional silencing. However, the methylation of CpG sites in regulatory regions outside the gene promoter also plays a role in regulating gene expression in a tissue-specific manner, thus making gene expression regulation through DNA methylation considerably more complex than simple promoter methylation. The process of gene silencing by DNA methylation is required for critical biological processes, such as cellular differentiation, genomic imprinting, X chromosome inactivation, and retrotransposon silencing. To date, two mechanisms have been proposed on how DNA methylation silences gene expression. The first mechanism posits that methylation prevents the binding of transcription factors to cytosine in the major DNA groove. The second mechanism suggests that methylation recruits proteins containing the methyl-CpG-binding domain to the 5mC, which in turn recruits certain histone modifiers, such as histone deacetylases, that change the chromatin state in a way that leads to a compact chromatin structure [6].

CpG methylation is mediated by the enzymatic family of DNA methyltransferases (DNMTs). In mammals, DNMTs use S-adenosyl methionine (SAM) as the methyl group donor to form the 5mC of the CpG. In humans, four main types of DNMTs exist, namely, DNMT1, DNMT3A, DNMT3B, and DNMT3L. DNMT1 is responsible for maintaining DNA methylation profiles during DNA replication in mitosis. DNMT1 recognises hemi-methylated CpGs and methylates them by copying the pre-existing DNA methylation profile from the parental DNA strands to the daughter DNA strands. DNMT3A and DNMT3B are considered *de novo* DNMTs. They recognise unmethylated CpGs and methylate them in response to various stimuli, thus creating new DNA methylation profiles. As such, DNMT3A and DNMT3B are responsible for establishing new DNA methylation profiles during early embryonic development and throughout postnatal life. DNMT3L, which functions in germ cells, enhances *de novo* methylation and increases the binding of methyl donors [6, 9, 10].

DNA demethylation can occur actively through deamination of the methylated or a nearby base through the action of activation-induced deaminase (AID), or through oxidising the methylated base through the action of ten-eleven translocation (TET) enzymes, both of which are independent of DNA replication. Base excision repair (BER) then replaces the modified nucleotide along with the surrounding nucleotides. DNA demethylation can also occur passively through diluting DNA methylation markers during DNA replication. However, the direct conversion of 5mC to cytosine does not occur [11].

## DNA methylation in T2DM

All the T2DM genetic risk factors identified by GWASs thus far have been estimated to account for only approximately 10% of the estimated heritability of T2DM. Therefore, research has continued to focus on exploring the role of epigenetics in T2DM in the hope of explaining some, if not all, of the missing heritability [6]. Recent studies have found that T2DM environmental risk factors regulate, through epigenetic modifications, the expression of T2DM genetic risk factors that control the particular intracellular signalling pathways involved in the onset and development of T2DM [2]. The following section focuses on what is known regarding the role of DNA methylation in the development and pathogenesis of T2DM, although the exact mechanisms underlying the involvement of DNA methylation in T2DM pathogenesis remain unclear [12].

## Single or multiple gene studies

### Pancreas and related cell lines

#### Insulin

Insulin is a peptide hormone secreted by pancreatic β cells upon nutrient uptake. It regulates blood glucose levels by enhancing glucose uptake and glycolysis in skeletal and adipose tissues [13]. During this process, insulin stimulates the translocation of the glucose transporter GLUT4 to the cell membrane from intracellular pools. Insulin also regulates blood glucose levels by enhancing the rate of glycogen synthesis in the liver, skeletal muscle, and adipose tissue, thus lowering the rate of glycogen breakdown in these tissues and inhibiting hepatic gluconeogenesis and glycogenolysis [14].

A number of *cis*-acting regulatory elements in the insulin promoter can work over large distances. These elements have been shown to bind a range of tissue-specific and ubiquitous transcription factors. One of these regulatory elements is the cyclic adenosine monophosphate (cAMP) responsive element (CRE), which can bind a diverse array of transcription factors [15]. In rodents, only one CRE site exists in the insulin promoter, whereas in humans, there are four, with CRE2 being the sole CRE conserved between the species. cAMP responsive element-binding protein-1 (CREB-1) and activating transcription factor-2 (ATF-2) are CRE-associated transcription factors. CREB-1 has an inhibitory effect on transcription, whereas ATF-2 activates transcription. Interestingly, CRE2 mutations and not CREB-1 mutations inhibited the ATF-2 effect [15]. The CRE site also plays a crucial role in insulin gene regulation [16].

The analysis of the human insulin (*INS*) and mouse *Ins2* promoters has identified the presence of nine and three CpG sites in the regions upstream of the transcription start site (TSS), respectively. Further analysis has revealed that the CpG sites present in the human *INS* and mouse *Ins2* promoters were differentially unmethylated in β cells compared with other tissues. Using the NIT-1 mouse insulinoma cell line, the methylation of these CpG sites has been shown to suppress the expression of the insulin-promoter-driven reporter gene by almost 90% [17].

To assess the role of individual methylation events on insulin gene expression, individual CpG sites were methylated in the mouse *Ins2* promoter and examined. The methylation of the CpG site within CRE was shown to independently suppress insulin promoter activity by approximately 50%. In addition, the methylation of one of the remaining two CpG sites had no effect on insulin gene expression, whereas the methylation of the other CpG site increased insulin gene expression by almost two folds. These findings indicate that methylation-dependent suppression of the insulin promoter is not simply additive, suggesting that other mechanisms are likely to cooperate with DNA methylation to suppress the expression of the insulin gene. Further analysis demonstrated that CRE CpG methylation inhibits the binding of CREB and ATF-2 to *Ins2* CRE in vivo (NIT-1 cells), but not in vitro, and increases the binding of methyl CpG binding protein 2 (MeCP2). These findings indicate that MeCP2 binds to the methylated promoter to inhibit the binding of insulin transcriptional activators, thus decreasing insulin gene expression [17]. An in vitro examination of *Ins2* in mouse embryonic stem cell cultures has previously indicated that the insulin gene is fully methylated; however, the gene becomes demethylated as the cells differentiate into insulin-producing β cells. Collectively, these findings suggest that the demethylation of the insulin promoter CpG sites plays a key role in β cell maturation and the tissue-specific expression of the insulin gene [17].

The role of DNA methylation was further examined in another study using human islets from T2DM and non-T2DM donors. The findings indicate that the glucose-stimulated insulin secretion (GSIS), insulin mRNA, and insulin content were reduced in the pancreatic islets of T2DM in comparison with the non-T2DM donors. In addition, four CpG sites (− 234, − 180, − 102, and + 63) showed increased DNA methylation in the T2DM pancreatic islets. Furthermore, insulin mRNA expression was found to negatively correlate with the degree of methylation in three of these CpG sites (− 234, − 180, and + 63). Researchers then investigated the effect of hyperglycaemia on the DNA methylation of the insulin promoter with clonal rat insulinoma-derived INS 832/13 β cells. When the cells were cultured under hyperglycaemic conditions (16.7 mmol/L glucose) for 72 h, the DNA methylation of two of the CpG sites (− 1057 and + 58) in the insulin gene promoter was found to be increased in β cells [18].

The effects of overnutrition on the DNA methylation status of the rat insulin-1 (*Ins1*) gene promoter were evaluated using a rat pancreatic insulin-producing β cell line (INS-1 cells) cultured under normal (11.2 mmol/L) or high (22.4 mmol/L) glucose concentrations for 14 days and Zucker diabetic fatty rats. The investigators found that the high glucose concentration increased *Dnmt1* mRNA expression levels and activity. This resulted in the increased DNA methylation of the five CpG sites within the *Ins1* promoter, including the CRE, and the suppression of *Ins1* mRNA expression, all in a glucose concentration and time-dependent manner [12]. In addition, increased *Ins1* promoter methylation was observed in pancreatic islets isolated from the Zucker diabetic fatty rats. Furthermore, insulin promoter-driven reporter gene (luciferase) activity was significantly suppressed by the artificial methylation of the *Ins1* promoter. *Ins1* mRNA suppression by high glucose concentration was significantly improved by a DNA methylation inhibitor. Moreover, the experimental high glucose conditions were found to significantly decrease TET activity and increase DNA methyltransferase activity. Metformin, the first line of T2DM treatment, has been shown to significantly suppress insulin promoter DNA methylation and upregulate *Ins1* mRNA expression [12]. Studies have also found reduced DNA CpG methylation at the *Ins1* promoter during the trans differentiation of fibroblasts, pancreatic exocrine cells, and WB-F344 (WB) rat liver epithelial cells to β cells [2].

#### Aristaless-related homeobox

The aristaless-related homeobox (*ARX*) gene is an X-linked transcription factor that functions in humans and mice to regulate the development of the pancreas among various other tissues. In the mouse pancreas, *Arx* has been found to be expressed throughout all stages of pancreatic development [19]. As such, *Arx* is first observed in the pancreatic anlage, followed by the differentiating precursors of the pancreatic endocrine cells and, finally, the ensuing α cells [20]. *Arx* deficiency in mice causes the loss of mature endocrine α cells, with a concomitant elevation in the numbers of δ cells and β cells [19] to maintain the total mass of the endocrine portion of the pancreas [21]. Humans with *ARX*-null mutations display a loss of α cells, indicating the essential role of *ARX* in mammalian α cell specification and differentiation [20].

A study, using a mouse pancreatic α cell line (α-TC1) and a mouse pancreatic insulinoma β cell line (Min6), was conducted to investigate the methylation of the CpG sites within α cells and β cells, respectively. The CpG-rich sites in the regulatory region of the lineage determination of the *Arx* gene were divided into two main groups, namely, UR1 (CpG-rich sites within the proximal promoter and close to the TSS) and UR2 (CpG-rich sites 2 Kb upstream of the TSS). The authors found that the majority of the CpG sites within the UR2 region were methylated in the β cells but remained unmethylated in the α cells [22].

Furthermore, in studies using mice and the Cre/loxP system, *Dnmt1*-deficient β cells have been shown to gradually convert to α cells. This alteration appears to be associated with the *Dnmt1* small interfering RNA (siRNA) on Min6 cells, along with the methylation status of the *Arx* UR2 region. This region is normally methylated (i.e., silenced *Arx*) in mature β cells but becomes hypomethylated (i.e., expressed *Arx*) in mature α cells and mature *Dmnt1*-deficient β cells. The methylated *Arx* UR2 region has been found to bind to MeCP2, which then recruits more proteins, such as Prmt6, a histone H3R2 methylase, to further suppress *Arx* expression. Although the methylation status of *Arx* has not yet been definitively linked with T2DM pathogenesis, an association has been suggested [22].

#### Pancreatic and duodenal homeobox-1

The pancreatic and duodenal homeobox-1 (*PDX-1*) is a transcription factor necessary for the differentiation of all pancreatic cell lineages. *PDX-1* expression is maintained at low levels in exocrine cells, where its function is yet to be fully understood. It is expressed at high levels in β cells, where it promotes the transcription of the insulin gene (i.e., insulin synthesis) [23].

The DNA methylation and mRNA expression levels of the *PDX-1* gene appear to play a crucial role in the differentiation of β cells. This process was analysed in the clonal rat insulinoma-derived INS 832/13 β cell line and human pancreatic islets from T2DM and non-T2DM donors. In comparison with non-T2DM, T2DM-derived pancreatic islets were shown to have a reduced *PDX-1* mRNA expression. This reduction was considered to be the result of the hypermethylation of 10 CpG sites located in the *PDX-1* distal promoter and enhancer regions. Accordingly, the reduction in *PDX-1* mRNA was positively correlated with GSIS and insulin mRNA expression. Furthermore, the hypermethylation of the CpG sites was negatively correlated with the *PDX-1* expression, a finding supported by the reduced expression of the reporter gene in clonal β cells. Although the data indicated that the hypermethylation in β cells from both humans and the cell line was due to hyperglycaemia, only in the cultured β cell line was the hyperglycaemia associated with significant increase in DNMT1 expression [24].

#### Glucagon-like peptide-1 receptor

Glucagon-like peptide-1 receptor (*GLP-1R*) is widely distributed in pancreatic islets [25]. The activation of GLP-1R in the β cells serves to enhance GSIS in a short-term manner. Although it cannot be directly monitored in humans, the continuous activation of GLP-1R is believed to increase β cell neogenesis and proliferation and overall insulin synthesis [26].

T2DM studies in humans and rats have indicated reduced *GLP-1R* expression in pancreatic islets, although whether this reduction is due to a change in the DNA methylation status of the *GLP-1R* gene and/or other mechanisms was initially unclear. Further analysis using pancreatic islets from T2DM and non-T2DM human donors was conducted to determine the mRNA expression levels of *MECP2*, *DNMT1*, *DNMT3A*, and *DNMT3B*, along with the DNA methylation status of 12 CpG sites (five upstream and seven downstream) associated with the *GLP-1R* TSS. Two of these CpG sites, at positions + 199 and + 205 bp from the TSS, were further analysed as a single CpG unit due to *GLP-1R* sequence characteristics [25]. This analysis showed decreased GSIS and reduced *GLP-1R* expression in pancreatic islets from the T2DM donors compared to non-T2DM donors. In addition, the CpG unit had a small increase in the level of DNA methylation in the T2DM islets compared with the non-T2DM islets. However, this increase was considered too small to persist after correction for multiple testing, and it did not demonstrate a significant correlation with *GLP-1R* mRNA expression. A trend toward reduced *DNMT3A* expression in the T2DM islets compared with the non-T2DM islets was also suggested. However, no difference was found between the T2DM and non-T2DM islets in terms of *MECP2*, *DNMT1*, and *DNMT3B* expression levels [25].

Furthermore, the DNA methylation status of the *GLP-1R* promoter was analysed using isolated α cells and β cells from the pancreatic islets. The DNA methylation level of the *GLP-1R* CpG site at position − 376 was significantly higher in α cells than in β cells, and this was found to be inversely correlated with the *GLP-1R* expression [25].

#### Peroxisome proliferator-activated receptor gamma coactivator-1 alpha

The peroxisome proliferator-activated receptor gamma coactivator-1 alpha (*PPARGC1A*) is a transcriptional coactivator of various transcription factors and nuclear receptors [27], including the peroxisome proliferator-activated receptor gamma (PPARG or PPARγ) [28]. *PPARGC1A* controls the activity of a wide range of transcription factors functioning in various cellular and metabolic processes, such as glycogenolysis, gluconeogenesis, fatty acid oxidation, oxidative phosphorylation, and glucose transport [29]. *PPARGC1A* is expressed predominantly in tissues with high energy demand including the skeletal muscle, pancreas, and liver [28].

A study was conducted to investigate whether the expression of *PPARGC1A* gene is altered in T2DM pancreatic islets and whether this alteration (if present) is due to DNA methylation (among other factors). In addition, the effect of the experimental downregulation of *PPARGC1A* on insulin secretion in human islets was investigated using human islets from T2DM and non-T2DM organ donors. The findings show that *PPARGC1A* mRNA expression was significantly reduced in T2DM islets compared with the non-T2DM islets. Furthermore, this study found a two-fold increase in the DNA methylation of the *PPARGC1A* promoter (four CpG sites analysed) in the T2DM islets, with a trend towards an inverse correlation between the level of *PPARGC1A* mRNA and *PPARGC1A* promoter methylation expression. In addition, the reduced *PPARGC1A* mRNA expression in the T2DM islets was positively correlated with reduced GSIS in the islets. These findings were confirmed by the experimental downregulation of *PPARGC1A* expression in human islets by siRNA, which resulted in the reduced expression of insulin mRNA and reduced insulin secretion, thus demonstrating a link between *PPARGC1A* expression level and GSIS [30].

### Blood

#### FOS-like antigen 2

In mice osteoblasts, the transgenic expression of *FOS-like antigen 2 (Fosl2)* leads to decreased body weight and increased bone mass, along with decreased serum levels of glucose, improved insulin sensitivity, and improved glucose tolerance. These findings indicate that *Fosl2 expression* plays a role in the positive regulation of insulin and glucose metabolism [29]. A detailed investigation into the blood DNA methylation status and expression level of the *FOSL2* gene found that eight CpG units within the FOSL2 gene had higher methylation rates in T2DM patients, resulting in a significant reduction in *FOSL2* mRNA and protein levels compared with normal glucose tolerance (NGT) group [31].

#### Protein tyrosine-protein phosphatase non-receptor type 1

Protein tyrosine phosphatases non-receptor type 1 (*PTPN1*) inactivates the transduction of the insulin signal cascade by dephosphorylating phosphotyrosine residues in insulin-signalling molecules [32]. Research into the association between T2DM susceptibility and the DNA methylation status of the *PTPN1* gene found a significant correlation between the risk for T2DM and the hypermethylation of the eight CpGs in the *PTPN1* promoter. A further breakdown analysis by gender showed that this correlation was female specific, as no significant differences were observed between T2DM males and control males in terms of *PTPN1* methylation [33].

#### Transcription factor 7-like 2

The transcription factor 7-like 2 (*TCF7L2*) gene plays an important role in various processes, including the activation of target genes in the Wnt signalling pathway [34], pancreatic β cell proliferation, and glucose homeostasis [35]. In addition, *TCF7L2* is the most significant and consistently replicated gene associated with an increased risk of T2DM [34]. Certain *TCF7L2* SNPs have been shown to be associated with increased T2DM risk [35]. Investigation into the DNA methylation profile of the *TCF7L2* gene promoter found a correlation between the methylation of specific CpGs, fasting glucose, total cholesterol, and LDL cholesterol. In addition, 13 out of the 22 *TCF7L2* promoter CpGs analysed were differentially methylated in the T2DM group compared with the control. However, the overall methylation pattern of the *TCF7L2* promoter did not show a clear differential pattern related to T2DM [36].

#### Solute carrier family 30 member 8

Pancreatic β cells are rich in zinc, which is essential for the zinc–insulin crystallisation that occurs within insulin-secretory vessels [37]. Solute carrier family 30 member 8 (*SLC30A8*) encodes the ZnT 8 (ZnT-8) protein, which is expressed in the insulin secretory granules of pancreatic β cells. ZnT-8 co-localises with insulin granules in the INS-1 pancreatic β cell line [38]. Genetic variation in the *SLC30A8* gene is associated with increased T2DM risk [39]. The genetic association between the *SLC30A8* gene and the increased risk of T2DM and diabetic nephropathy (DN) was investigated, by examining the DNA methylation status of six CpG sites in *the SLC30A8* promoter using blood samples. The methylation level of the *SLC30A8* promoter was found to be higher in T2DM patients compared with NGT individuals. There was no significant difference in DNA methylation between T2DM with or without DN[40].

#### Insulin-like growth factor-1

Insulin-like growth factor-1 (*IGF-1*) is involved in metabolism of carbohydrates, proteins, and lipids. Furthermore, IGF-1 has known insulin-like effects, as it has been experimentally demonstrated that the administration of recombinant human IGF-1 improves insulin sensitivity and reduces blood glucose levels in patients with T2DM [41]. Research into the DNA methylation status of *IGF-1* using blood samples of T2DM found one CpG site with increased DNA methylation in the T2DM patients. Moreover, IGF-I serum levels were reduced in the T2DM patients compared with the NGT individuals [42].

#### Insulin-like growth factor-binding protein 1

Insulin-like growth factor-binding protein 1 (*IGFBP-1*) is believed to play a role in glucose metabolism through its regulatory effect on IGF-1. Evidence has suggested an inverse correlation between endogenous IGFBP-1 levels and free IGF-I levels, with the injection of exogenous IGFBP-1 serving to reduce circulating glucose and free IGF-I levels. Furthermore, the insulin resistance state is associated with the reduced synthesis and circulating level of IGFBP-1. Free IGF-I levels have been shown to be elevated, whereas IGFBP-1 levels are reduced in patients with T2DM [43]. Analysis of DNA methylation status of *IGFBP-1* and its association with serum *IGFBP-1* levels in T2DM found that the DNA methylation levels of six CpG sites were higher in T2DM patients compared with the control individuals. Newly diagnosed patients with T2DM with a familial history of the disease showed higher *IGFBP-1* DNA methylation levels compared with those without a familial history of the disease. These findings suggest that increased *IGFBP-1* DNA methylation levels and decreased IGFBP-1 serum levels are features of newly diagnosed T2DM [44].

#### Insulin-like growth factor-binding protein 7

Insulin-like growth factor-binding protein 7 (*IGFBP-7*) has low affinity for IGF-1, but binds insulin with a higher affinity. A study found significantly greater IGFBP-7 serum levels in T2DM patients undergoing haemodialysis compared with non-diabetic haemodialysis patients [45]. Studies on the DNA methylation status of *IGFBP-7* gene and its association with serum levels in T2DM found that three CpG sites had increased DNA methylation in men newly diagnosed with T2DM compared with control individuals. However, IGFBP-7 serum levels were similar among all the groups (newly diagnosed T2DM, treated T2DM, and control subjects) and did not show a correlation with *IGFBP-7* DNA methylation levels. In addition, IGFBP-7 serum levels were positively correlated with the serum levels of IGFBP-1, which has been shown to be an insulin production marker, in T2DM newly diagnosed men but not women. Thus, the authors concluded that low IGFBP-7 may be associated with T2DM insulin resistance [46].

#### B cell CLL/lymphoma 11A

B cell CLL/lymphoma 11A (*BCL11A) gene* is believed to be involved in various important processes, including neuronal regulation of hormonal secretion, cell–cell signal transduction [47], expression of human foetal haemoglobin, negative regulation of p53 activity, and lymphopoiesis [48]. A GWAS conducted in non-diabetic participants found that rs243021 SNP in BCL11A gene is associated with reduced insulin secretion and increased fasting glucagon levels, suggesting an increased risk of T2DM [47]. The findings of this study are in agreement with those of a previous study where SNPs in five loci, representing ADAM metallopeptidase with thrombospondin type 1 motif 9 (*ADAMTS9*, rs4607103), *BCL11A* (rs10490072), cell division cycle protein 123/calcium/calmodulin-dependent protein kinase 1D (*CDC123/CAMK1D*, rs12779790), melatonin receptor 1B (*MTNR1B*, rs10830963), and THADA armadillo repeat containing (*THADA*, rs7578597), were found to affect β cell function [49].

In another study, the correlation between the DNA methylation of five CpG sites within an intragenic CpG island in the *BCL11A* gene and T2DM was studied. No significant association was found between *BCL11A* DNA methylation and either T2DM or non-T2DM individuals. However, when the association analysis was broken down by gender, a significant association between *BCL11A* DNA methylation and T2DM in males was identified. In addition, a substantial correlation between the mean level of DNA methylation in *BCL11A* and triglyceride levels in females was found. These findings suggest that *BCL11A* DNA methylation may directly contribute to T2DM risk in males but may contribute indirectly to T2DM risk in females through its influence on triglyceride metabolism [48].

#### Glucokinase

The glucokinase (*GCK)* gene is predominantly expressed in the liver and, to a lesser extent, in the pancreas [50]. Studies have found that one of the main roles of insulin is to stimulate *GCK* expression to activate relevant glycolytic genes, thus increasing glucose utilisation. Indeed, insulin resistance due to downregulated glucokinase activities in patients with T2DM has been reported, indicating that *GCK* is a T2DM susceptibility gene [51].

Whether the degree of DNA methylation in 11 CpG sites of the hepatic *Gck* promoter influences its expression and activity and whether age influences its expression and age-related diabetes were investigated in Wistar rats. The results demonstrate an age-associated reduction in hepatic *Gck* mRNA expression and glucokinase activity, with an increasing decline with increasing age. Furthermore, the 11 CpG sites analysed showed age-related progressive methylation changes. Treatment with the DNA methyltransferase inhibitor 5-aza-2’-deoxycytidine (5-Aza-CdR) increased *Gck* expression in the rat primary hepatocytes by fourfold. The study also demonstrated that the age-related increase in hepatic *Gck* DNA methylation was negatively associated with its expression, suggesting that DNA methylation may have a role in increasing age-dependent susceptibility to hepatic insulin resistance, ultimately leading to diabetes [51].

Further research was conducted to investigate the correlation between the DNA methylation of four CpG sites within an intragenic CpG island in the *GCK* gene and T2DM. Only CpG4 had increased DNA methylation in T2DM patients compared with the controls. When the association analysis was broken down by gender, it was found to be male specific. In addition, the authors found that the increased CpG4 DNA methylation was also associated with higher total cholesterol concentration in a male-specific manner. The study concluded that the increased CpG4 DNA methylation may contribute to the increased T2DM risk in males [52].

#### Monocyte chemoattractant protein-1

Monocyte chemoattractant protein-1 (*MCP-1*) is a chemokine essential for regulating the migration and infiltration of monocytes, natural killer cells, macrophages, and memory T lymphocytes. *MCP-1* can be produced either constitutively or in response to cytokines, oxidative stress, or growth factors [53]. A study was conducted to investigate variations in MCP-1 protein serum levels in patients with DM and metabolic syndrome (MetS) and found the serum MCP-1 protein levels were significantly higher in the MetS group. In addition, the DM group had higher MCP-1 levels compared with the MetS group. A correlation analysis found a significantly positive correlation between MCP-1 and waist circumference, the waist–hip rate, body mass index (BMI), triglyceride levels, HOMA-IR, systolic blood pressure, and diastolic blood pressure [54].

As a follow-up, a study was conducted to investigate the blood DNA methylation status of MCP-1 promoter CpG sites and their association with serum MCP-1 and blood glucose levels in patients with T2DM and healthy (control) individuals. The MCP-1 promoter was found to be methylated in 8 out of 32 patients with T2DM and 12 out of 15 control individuals, suggesting that the *MCP-1* promoter region was methylated primarily in control individuals compared with the patients with T2DM. In addition, the DNA methylation levels of the *MCP-1* promoter correlated negatively with MCP-1 serum levels, fasting blood glucose, glycated haemoglobin A1c (HbA1c), triglyceride levels, and BMI. The study concluded that blood glucose and triglyceride levels may contribute to the hypomethylation of *MCP-1* promoter CpG sites, resulting in increased MCP-1 serum levels [55].

#### Breast cancer 1 DNA repair associated, peroxiredoxin 2, tumour protein p53 and scavenger receptor class A member 3

In an attempt to investigate the role of DNA methylation in the pathogenesis and progression of T2DM, a study analysed the blood DNA methylation changes in 22 genes known to be involved in cellular stress and toxicity, along with the expression levels of methyl-CpG-binding domain protein 2 *(MBD2)* as a marker of DNA methylation. The study found that *MBD2* mRNA expression levels were higher in T2DM patients compared with control individuals. In addition, the authors found that increased T2DM duration was associated with a consistent increase in the DNA methylation fraction of certain tumour suppressor genes, including tumour protein p53 (*TP53)* and breast cancer 1 DNA repair associated (*BRCA1*), along with the oxidative stress protection genes scavenger receptor class A member 3 *(SCARA3*) and peroxiredoxin 2 (*PRDX2)*. This hypermethylation suggested a possible underlying mechanism for increased cancer risk and the disruption of oxidative stress protection in patients with T2DM. The study concluded that the increased *MBD2* mRNA expression in the T2DM patients may be responsible for the general dysregulation of DNA methylation resulting from the disease [56].

#### Protein kinase C epsilon zeta

The protein kinase C epsilon zeta (*PRKCZ*) is believed to be involved in the translocation of the *GLUT4* protein and in the insulin-signalling pathway downstream of PI 3-kinase. Certain SNPs in *PRKCZ* gene were shown to be associated with T2DM risk in Han Chinese population [57]. A study was conducted to assess the correlation between T2DM and the DNA methylation of nine CpG sites in the promoter of the *PRKCZ* gene. The authors found that T2DM patients had higher DNA methylation levels compared with controls. Furthermore, T2DM patients had reduced PRKCZ serum levels, indicating reduced expression. These findings suggest that *PRKCZ* hypermethylation may be involved in the pathogenesis of T2DM [58].

#### Gastric inhibitory polypeptide receptor

Gastric inhibitory polypeptide (*GIP*) is an incretin gastrointestinal hormone that function in stimulating the insulin response following an oral glucose challenge. Studies have found that the GIP action is reduced in patients with T2DM, although its secretion seems to be normal. Studies have also demonstrated the role of the gastric inhibitory polypeptide receptor (*GIPR*) in promoting the function and survival of pancreatic β cells. Therefore, *GIPR* may play a role in the mediation of insulin secretion following an oral glucose challenge [59]. A study was performed based on the hypothesis that altered *GIPR* DNA methylation profiles may be involved in the reduced GIP action observed in patients with T2DM. The study evaluated differences in DNA methylation of 13 CpG sites of the *GIPR* promoter using blood. The authors found that *GIPR* promoter was hypomethylated in patients with T2DM compared to the control individuals. The mean methylation levels of the *GIPR* promoter were negatively correlated with fasting glucose and HOMA-IR in the patients with T2DM, where the reduced DNA methylation of the *GIPR* promoter was associated with increased HOMA-IR and increased fasting glucose levels [60].

#### Calmodulin 2, calmodulin-dependent protein kinase 1D*, and* cryptochrome circadian regulator 2

Calmodulin 2 (*CALM2)* functions in Ca^2+^ sensing and signal transduction [61]. A specific polymorphism in *CALM2 (*rs815815*)* has been associated with the dialysis survival of African-American patients with T2DM-associated end-stage renal disease [62]. The calmodulin-dependent protein kinase 1D (*CAMK1D*) gene is a member of the Ca^2+^/calmodulin-dependent protein kinase family, which relays important intracellular calcium signals required for various cellular processes. When calcium influx occurs in hippocampal neurons and granulocyte cells, *CAMK1D* activates CREB-dependent gene transcription. Considering the role of CREB in β cell survival, along with the role of cytosolic calcium in regulating the exocytosis machinery in β cells, CAMK1D may play a role in β cell insulin secretion [63].

Studies have found that mice carrying null mutations in cryptochrome circadian regulator 2 (*Cry2)* gene have a number of metabolic abnormalities, including increased insulin sensitivity, impaired glucose tolerance, reduced adipose tissue and body weight, and abnormal circadian rhythmicity. A meta-analysis of 21 GWASs investigating fasting insulin, fasting glucose, HOMA-IR, and homeostatic model assessment of β cell function (HOMA-B) identified several loci, one of which was a particular locus in or near *CRY2 (*rs11605924), that was associated with fasting glucose [64]. A study investigated the contribution of promoter DNA methylation of *CALM2* (four CpG dinucleotides), *CAMK1D* (nine CpG dinucleotides), and *CRY2* (five CpG dinucleotides) to T2DM risk. The study concluded that these three genes do not contribute to T2DM risk [65].

### Multiple organs/tissues

#### Protein tyrosine phosphatase receptor type D

The protein tyrosine phosphatases receptor type D (*PTPRD*) catalyses the hydrolytic removal of phosphate group(s) from the tyrosine residues of target proteins. This reaction is crucial because the phosphorylation status of protein tyrosine residues is considered an important cellular signal transduction mechanism [66].

A GWAS was conducted to investigate T2DM susceptibility genes in a Han Chinese population. The study identified, a specific SNP (rs17584499) in *PTPRD* that was associated with increased T2DM risk in the population studied [67]. A replication study of the previous GWAS was conducted to identify the mechanism by which the *PTPRD* SNP (rs17584499) influences glucose homeostasis. The results of the study revealed that *PTPRD* SNP (rs17584499) was associated with increased in HOMA-IR over time. The expression of *PTPRD* in adipose tissue negatively associated with HOMA-IR and fasting insulin levels, thus facilitating the progression to T2DM [68]. Furthermore, another SNP in *PTPRD* (rs649891) that has been associated with increased T2DM risk in a Mexican population was identified by another GWAS and meta-analysis [69]. However, the exact mechanism by which PTPRD interacts with the insulin-signalling pathway and increases T2DM risk and whether epigenetics is involved remain unclear [70].

As a follow-up study, an evaluation of DNA methylation-induced changes in the *PTPRD* expression in T2DM and non-T2DM individuals was conducted. This study used blood samples from patients with T2DM and healthy (control) individuals, along with the human liver carcinoma cell line HepG2 and liver tissue from male KK and KK-Cg-*A*^*y*^/J mice of three age groups. The age groups of the mice consisted of 6 weeks old (representing the early disease stage), 16 weeks old (representing the middle disease stage), and 42 weeks old (representing the late disease stage). The study found that the *PTPRD* mRNA expression level was lower in patients with T2DM compared with that in the controls. In addition, the *PTPRD* mRNA expression was correlated with the duration of T2DM in these patients, in whom there was a general decrease in the *PTPRD* mRNA expression with increasing T2DM duration. When the human *PPARG2* was overexpressed in HepG2 cells, it induced the insulin receptor and *PTPRD* overexpression. When *PTPRD* was knocked down, the insulin receptor was downregulated, indicating that *PTPRD* may be involved in the insulin-signalling pathway through *PPARG2* [70].

The study also demonstrated that the expression of the *Ptprd* protein was significantly reduced in late-stage diabetic mice compared with the early-stage, middle-stage, and control mice. Similar to humans, the mRNA expression level of *Ptprd* decreased with the increasing disease stage (duration), and the *Ptprd* promoter was found to be hypermethylated in the middle- and late-stage diabetic mice but not early-stage mice. Further analysis revealed that the *Dnmt1* expression was significantly higher in the late-stage mice. In contrast to the results obtained in the mice, patients with T2DM from all disease durations showed *PTPRD* promoter hypermethylation. In addition, the *DNMT1* mRNA expression was found to be higher in patients with T2DM compared with the controls. Collectively, the findings of the study suggest that DNMT1 caused *PTPRD* DNA hypermethylation and silenced insulin signalling in patients with T2DM [70].

#### Growth factor receptor-bound protein-10

The growth factor receptor-bound protein-10 (*GRB10*) is an adapter protein that functions as a down regulator of the insulin receptor through its interaction with various signalling molecules and receptor tyrosine kinases, thus it has been implicated in T2DM pathogenesis. *GRB10* is expressed in a variety of tissues, with the highest expression in pancreas. In addition, *GRB10* is imprinted in a parent-of-origin manner in various tissues [71].

A large-scale meta-analysis for GSIS during oral glucose tolerance test (OGTT) was conducted on non-diabetic individuals. The analysis identified impaired β cell function-associated variants in the *GRB10* gene. The study then investigated the contribution of *GRB10* risk alleles to T2DM risk, along with their transmission patterns and the effect they may have on glucose and insulin levels during OGTT. The methylation status and gene expression of *GRB10* was also analysed in human pancreatic islets from T2DM and non-T2DM donors and peripheral blood lymphocytes from T2DM. The mechanisms of GRB10 effect on pancreatic α and β cell functions was also analysed in human pancreatic islets and in rat insulinoma INS-1 cells [71].

Interestingly, the study associated a specific *GRB10* variant, namely, the A-allele of rs933360, with decreased insulin sensitivity, non-affected GSIS, increased fasting plasma glucose level and increased risk of T2DM if inherited from the father and an enhanced insulin sensitivity, reduced GSIS, and reduced fasting plasma glucose level if inherited from the mother. The results also show an allelic imbalance and tissue-specific differences in *GRB10* DNA methylation levels in the human pancreatic islets compared to the peripheral blood lymphocytes with higher *GRB10* DNA methylation in human pancreatic islets. The disruption of *GRB10* in human pancreatic islets resulted in reduced insulin and glucagon expression and secretion and significant reduction in the number of viable pancreatic islets. The disruption of *Grb10* in rat insulinoma INS-1 832/13 cell line resulted in reduced GSIS. Taken together, these findings suggest that *GRB10* tissue-specific DNA methylation, within the context of imprinting, contributes to T2DM pathogenesis through the modulation of glucose metabolism, placing more emphasis on the need to investigate whether T2DM-associated risk alleles are maternally or paternally inherited [71].

#### *Peroxisome proliferator-activated receptor gamma* and *pyruvate dehydrogenase lipoamide kinase isozyme 4*

The ability of a system to alternate between glucose and lipid oxidation as a fuel source based on the nutrient availability is termed as metabolic flexibility. Failure of metabolic flexibility is often accompanied by a number of symptoms such as insulin resistance. The competition between glucose and fatty acid oxidation occurs at the pyruvate dehydrogenase complex (PDC) level, which is normally active during healthy and well-fed state. Pyruvate dehydrogenase kinase (*PDK*), of which *PDK4* is an isozyme, works to supress PDC when glucose is scarce, to maintain energy homeostasis. The inappropriate suppression of PDC activity may be involved in the development of metabolic diseases [72].

A study was conducted to investigate the DNA methylation of certain candidate genes and whether differential levels of DNA methylation in the blood could be used as biomarkers for T2DM and/or MetS. The candidate genes were fat mass and obesity-associated (*FTO)*, *KCNJ11*, potassium voltage-gated channel subfamily Q member 1 (*KCNQ1)*, *PDK4*, *PDX-1*, paternally expressed gene-3 (*PEG3)*, *PPARG,* and stearoyl-coenzyme A desaturase-1 (*SCD1)*. These genes were chosen based on their functional relevance to certain metabolic processes, including glucose metabolism, β cell maturation and pancreatic development, insulin secretion, adipogenesis, and fatty acid storage and metabolism, along with their potential contribution to the development of various metabolic diseases. Indeed, five of these candidate genes (*FTO, KCNQ1, PDK4*, *PDX-1*, and *PPARG*) have previously been identified to have differential DNA methylation levels in patients with T2DM. The study utilised peripheral blood leukocytes from three groups of individuals: those with T2DM and MetS, those with T2DM only and healthy (control) individuals. All of the patients diagnosed with T2DM and MetS (first group) had central obesity [73].

Although a trend toward significance was observed for *PEG3*, the study did not detect any significant differential gene-specific DNA methylation between the patients and controls for any of the other candidate genes. However, a trend toward a positive correlation was detected between HDL cholesterol levels, which can be downregulated by p53 and the methylation of the p53 mediator *PEG3*. When the patients were divided into T2DM and MetS subgroups and single CpG loci were analysed, differential DNA methylation levels between the groups were observed for 4 out of the 42 loci tested. The four CpG loci were found to be located within the promoters of *FTO*, *KCNJ11*, *PDK4*, and *PPARG* [73].

The authors of this study also found that the CpG locus in *KCNJ11* had significantly higher methylation levels in MetS patients compared with the patients with T2DM and controls. The authors speculated that the elevated DNA methylation in this locus may mimic genetic *KCNJ11* defects. In addition, the CpG locus in the *PPARG* promoter was found to have higher methylation levels in patients with T2DM compared with controls [73].

The study demonstrated that the differentially methylated CpG locus in *PDK4* also showed decreased methylation levels in T2DM and MetS patients compared with the control group [73]. This finding is consistent with a previous study that reported an increase in *PDK4* mRNA expression in the skeletal muscle of patients with T2DM due to *PDK4* promoter hypomethylation [74].

#### ATP-binding cassette subfamily G member 1, sterol regulatory element-binding transcription factor 1, phosphoethanolamine/phosphocholine phosphatase 1, and thioredoxin-interacting protein

ATP-binding cassette subfamily G member 1 (*ABCG1*) is a cholesterol transporter important for maintaining cellular cholesterol homeostasis in pancreatic β cells [75]. The phosphoethanolamine/phosphocholine phosphatase 1 (*PHOSPHO1*) gene encodes a hydrolase enzyme (phosphatase) that is involved in vascular and skeletal mineralisation. Indeed, T2DM is a common cause of cardiovascular calcification. As such, *PHOSPHO1* is a candidate marker for T2DM-linked CVD [76, 77]. Suppressor of cytokine signalling 3 (*SOCS3*) is a member of the intracellular, cytokine-inducible *SOCS* family that functions in regulating the JAK/STAT pathway in various cell types*.* SOCS3 acts as a regulator of insulin signalling [78].

The sterol regulatory element-binding transcription factor 1 (*SREBF1*) gene encodes the sterol regulatory element-binding transcription factor proteins SREBP-1a and SREBP-1c, which function in the regulation of fatty acid and cholesterol synthesis. The skeletal muscle and adipose tissue from patients with T2DM have demonstrated reduced SREBP-1c expression, suggesting a possible contribution of low levels of SREBP-1c towards insulin resistance. Furthermore, several studies have associated variations in the *SREBF1* gene with increased T2DM risk [79]. Thioredoxin-interacting protein (TXNIP) plays an important role in the regulation of the cellular redox status. Studies have shown that hyperglycaemia causes glucose toxicity (i.e., β cell dysfunction and apoptosis), with TXNIP considered a key mediator of these effects primarily through the mitochondrial death pathway. Moreover, *TXNIP* overexpression has been shown to cause β cell apoptosis. In DM, the *TXNIP* expression is increased in β cells, so increased *TXNIP* expression may be a key player in the pathogenesis of DM. In support of this notion, the deletion and mutation of *Txnip* have been found to protect against diabetes in a variety of T1DM and T2DM mouse models [80].

A nested case–control study investigated blood DNA methylation levels in Indian Asians (the discovery group) and Europeans (the replication group) to determine if there was an association with T2DM risk. The participants were identified from 25,372 participants in the 8-year follow-up London Life Sciences Prospective Population study (LOLIPOP). The result of the investigation reported five T2DM-associated DNA methylation CpG loci in blood DNA, namely, *ABCG1, PHOSPHO1, SOCS3, SREBF1*, and *TXNIP* [81].

A follow-up study was then conducted to examine the potential use of these five loci to predict the future occurrence of T2DM in *subjects from* the family-based Botnia prospective study. The study also investigated whether these five loci and, by extension, their associated genes, displayed altered DNA methylation and gene expression in the primary T2DM target tissues, including adipose tissue, pancreatic islets, liver and skeletal muscle, in T2DM and non-T2DM individuals. The study utilised blood samples from T2DM and non-T2DM participants from the Botnia prospective study, in addition to blood, skeletal muscle and adipose tissue from monozygotic twins discordant for T2DM, pancreatic islets from T2DM and non-T2DM donors and human liver DNA methylation data from the Kuopio Obesity Surgery Study (KOBS) [77].

The investigators found that the blood DNA methylation of the *PHOSPHO1* locus (cg02650017) was associated with a 15% decreased risk of T2DM, whereas the methylation of the *ABCG1* locus (cg06500161) was associated with a 9% increased risk for future T2DM. Blood DNA methylation at *SREBF1* (cg11024682), *TXNIP* (cg19693031), and *SOCS3* (cg18181703) was not associated with T2DM risk in subjects from the Botnia prospective study. In addition, the blood DNA methylation levels of the *PHOSPHO1* locus showed a positive correlation with HDL levels. *PHOSPHO1* DNA methylation level was decreased in the skeletal muscle from the T2DM subjects, although this decrease did not result in an altered gene expression. The blood DNA methylation of the *ABCG1* locus showed a positive correlation with fasting insulin, HbA1c, BMI, and triglyceride levels in the Botnia subjects. Moreover, *ABCG1* DNA methylation level was increased in the blood and adipose tissue of T2DM subjects, while its expression altered in muscle tissue from T2DM subjects [77].

In addition, the blood DNA methylation levels of the *SREBF1* locus (cg11024682) showed a positive correlation with age, BMI, fasting glucose, and HbA1c in subjects from the Botnia prospective study. Furthermore, *SREBF1* DNA methylation was increased in T2DM pancreatic islets, while its expression was found to be altered in a diabetic liver and muscle tissues. The blood DNA methylation of the *SOCS3* locus (cg18181703) showed a negative correlation with BMI and age. Moreover, *SOCS3* DNA methylation demonstrated a positive correlation with its expression in human pancreatic islets, but correlated negatively with its expression in the adipose tissue. The blood DNA methylation levels of the TXNIP locus (cg19693031) showed a negative correlation with triglyceride levels. In addition, *TXNIP* DNA methylation was found to be decreased in the blood, skeletal muscle, and pancreatic islets from the T2DM subjects, while its expression altered in the muscle tissue of subjects with T2DM [77]*.*

The authors also found that the blood DNA methylation levels of the CpG loci at *SOCS3* and *SREBF1* correlated with the degree of methylation in the adipose tissue. The authors concluded that DNA methylation biomarkers in blood might be used as surrogate biomarkers when the target tissue is inaccessible [77]. An epigenome-wide association study (EWAS) investigating the association between DM and blood DNA methylation in ischemic stroke patient cohorts found that cg19693031 CpG (TXNIP) DNA hypomethylation was associated with T2DM. The authors also found that TXNIP DNA methylation was inversely associated with HbA1c levels in patients with T2DM with a poor control of their blood glucose levels [82].

## Genome-wide methylation studies

### Pancreas

#### CpG-SNPs

Although previous studies have identified approximately 40 T2DM-associated SNPs, the exact mechanism by which these SNPs affect their target genes in a way that increases the risk of T2DM remains unclear. One suggested mechanism by which SNPs change gene expression is through epigenetics via the introduction or removal of CpG sites, which are potential DNA methylation sites. In addition, these CpG-SNPs may affect the expression of their target gene(s) through other mechanisms, such as interfering with the binding of certain proteins [83] or affecting intragenic DNA methylation through exonic splicing enhancers. Previous studies have shown that intragenic DNA methylation plays a key role in the regulation of alternative splicing [84].

Few studies have explored the role of CpG-SNPs in T2DM pathogenesis. A study was conducted to investigate whether any of the 40 previously reported T2DM-associated SNPs serve to introduce or remove potentially important DNA methylation sites, including CpG sites. The study also investigated whether these CpG-SNPs were associated with differential DNA methylation levels, which may induce changes in the hormone secretion of human pancreatic islets via changes in gene expression and/or alternative splicing events. The authors utilised human pancreatic islet tissue from non-T2DM donors [83].

The findings of the study indicated that 19 of the 40 (48%) T2DM-associated SNPs either introduced or removed a CpG site. Of these, four were determined to be coding SNPs, while five were considered intronic and the other ten were intergenic. DNA methylation data were successfully generated for 16 of the 19 CpG-SNP loci and identified the candidate genes, adenylate cyclase 5 (*ADCY5,* rs11708067*)*, cyclin-dependent kinase inhibitor 2A (*CDKN2A,* rs564398*)*, dual specificity phosphatase 8 (*DUSP8,* rs2334499*)*, dual specificity phosphatase 9 (*DUSP9,* rs5945326*)*, *PPARG (*rs1801282*)*, insulin receptor substrate 1 *(IRS1) (*rs7578326*)*, and wolframin ER transmembrane glycoprotein (*WFS1,* rs1801214*) (*CpG-SNP risk alleles which removed a CpG site*)*, CDK5 regulatory subunit-associated protein 1-like 1 (*CDKAL1,* rs7754840*)*, coiled-coil-helix-coiled-coil-helix domain-containing 2 pseudogene 9 (*CHCHD9,* rs13292136*)*, haematopoietically expressed homeobox (*HHEX,* rs5015480*)*, high-mobility group AT-hook 2 (*HMGA2,* rs1531343*)*, *KCNQ1 (*rs2237895*)*, *SLC30A8 (*rs13266634*)*, serine racemase (*SRR,* rs391300*)*, *TCF7L2 (*rs7901695*)*, and tetraspanin 8 *(TSPAN8,* rs7961581*) (*CpG-SNPs which introduced a CpG site*)* in human islets. DNA methylation data were then successfully generated for a total of 22 CpG sites located near *ADCY5*, *CDKN2A*, *DUSP9*, *HHEX*, *HMGA2*, *KCNQ1*, *PPARG*, *SLC30A8*, *TCF7L2*, and *WFS1*, all of which were found to be T2DM-risk SNPs. However, DNA methylation data could not be generated for the remaining three T2DM-associated CpG-SNPs, namely, *CDC123 (*rs12779790*), KCNJ11 (*rs5219*), and* zinc finger BED-type containing 3 *(ZBED3,* rs4457053) due to design/technical issues [83].

From this study, all of the 16 T2DM-associated CpG-SNPs that generated successful data were associated with CpG-SNP sites of differential DNA methylation in the human islets. In addition, six CpG-SNP loci, representing *ADCY5*, *CDKN2A*, *HMGA2*, *KCNQ1*, *TCF7L2*, and *WFS1*, were associated with differential DNA methylation of the surrounding CpG sites. Furthermore, some of the differentially methylated T2DM-associated CpG-SNP sites in the study were associated with gene expression, alternative splicing events, and hormone secretion in human islets. For example, carriers of the CpG-SNPs rs391300 (*SRR*) and rs5945326 (*DUSP9*), which showed increased DNA methylation, had decreased mRNA expression of replication protein A1 (*RPA1*) and arginine vasopressin receptor 2 *(AVPR2*), respectively, in human islets [83].

Furthermore, the T2DM-associated CpG-SNPs located in *CDKAL1, SLC30A8*, *TCF7L2,* and *WFS1* were found to be associated with alternative splicing events in their respective genes in human islets. Moreover, some of the differentially methylated T2DM-associated CpG-SNP sites were found to be associated with hormone secretion in the human islets, where CpG-SNPs in *ADCY5* (rs11708067) and *KCNQ1* (rs2237895) were associated with glucagon secretion, whereas those of ADCY5 (rs11708067) and *HHEx* (rs5015480) were associated with insulin secretion, and that of *CDKN2A* (rs564398) was associated with insulin content. The study also found that the 19 identified T2DM-associated CpG-SNP sites were in strong linkage disequilibrium with 295 other SNPs, 91 of which were CpG-SNPs. Collectively, the study suggests that the introduction and/or removal of CpG sites should be considered a molecular mechanism through which T2DM-associated SNPs may contribute to the pathogenesis of T2DM [83].

Similarly, another study was conducted to investigate the association between a particular *WFS1* CpG-SNP (rs1801214-T allele) located in the coding sequence and T2DM risk in an Iranian population. The study utilised blood samples and found that this specific CpG-SNP, which removes a CpG site, was significantly associated with lower T2DM risk in this population. However, the authors did not perform any measurements of *WFS1* expression, nor did they investigate its methylation status [85].

#### Cyclin-dependent kinase inhibitor 1A, Exocyst complex component -3 like 2, Phosphodiesterase-7B, and Septin-9

A genome-wide DNA methylation profiling analysis was performed to assess the human pancreatic islet DNA methylome and the epigenetic basis of T2DM pathogenesis. The study assessed the transcriptome and DNA methylation of 479,927 CpG sites utilising pancreatic islets from T2DM and non-diabetic donors. They also utilised INS-1 832/13 β cells and αTC 1-6 cells for expression and functional analyses. A total of 1649 CpG sites were identified to be differentially methylated in the T2DM islets with ≥ 5% differences in methylation, of which 561 were considered intergenic, and 1008 were located near or in 853 genes. Of the 853 genes, 102 were found to be differentially expressed in the T2DM islets [86]. In addition, 26 genes (~ 25%) were considered to be positively correlated with DNA methylation, where DNA hypomethylation was associated with reduced gene expression. By contrast, 77 genes (~75%) had an inverse relationship with DNA methylation, where DNA hypomethylation was associated with elevated gene expression. The authors then performed KEGG pathway analysis on these 853 differentially expressed genes and identified MAPK signalling pathway, pathways in cancer, ECM-receptor interaction, focal adhesion, and axon guidance, and regulation of actin cytoskeleton as some of the pathways involved [86].

The authors identified 75 genes exhibiting decreased DNA methylation and increased gene expression in T2DM pancreatic islets compared to non-diabetic islets. The authors then selected three of these genes and overexpressed them in clonal β cells, to gain better understanding on the mechanisms by which these genes may contribute to impaired β cell function and T2DM development and to model the situation in humans. The genes septin-9 (*Sept9*), cyclin-dependent kinase inhibitor 1A (*Cdkn1a*), and phosphodiesterase-7B (*Pde7b*) were selected based on their potential role in islet function and DM, along with their differential DNA methylation in multiple CpG sites and differential expression in T2DM islets. Overexpression of *Sept9* had no significant effect, while overexpression of *Pde7b* and *CDKN1A* significantly reduced GSIS in clonal β cells. Moreover, increased *Cdkn1a* expression decreased β cell proliferation. Then, the authors overexpressed these three genes in clonal α cells to examine the effect on α cells. Overexpression of *Sept9* produced a significant increase in glucagon expression, while overexpression of *Pde7b* and *Cdkn1a* increased glucagon release [86].

In this study, exocyst complex component 3-like 2 (*EXOC3L2*) demonstrated increased DNA methylation and reduced expression in T2DM islets compared to non-diabetic islets. The authors silenced *Exoc3l2* in clonal β cells, to model the decreased *EXOC3L2* expression in human T2DM islets, and to examine the effect on β cell exocytosis. The authors found that silencing *Exoc3l2* reduced β cell exocytosis. The authors concluded that the findings of this study can serve as a reference for human pancreatic islets methylome [86].

#### Solute carrier family 2 member 2, suppressor of cytokine signalling 2, Parkin RBR E3 ubiquitin protein ligase, nuclear receptor subfamily 4 group A member 3, and phosphotyrosine interacting domain-containing 1

In humans, mutations in solute carrier 2 member 2 (*SLC2A2*, also known as GLUT2) have been associated with autosomal recessive neonatal diabetes [87]. GWASs have found several *SLC2A2* variants that are associated with an increased risk of various disorders, including T2DM, cardiovascular disease, and hypercholesterolemia [88]. Parkin RBR E3 ubiquitin protein ligase (*PARK2*) has been linked to several diseases, including T2DM [89]. Certain SNPs in *PARK2* have been shown to be associated with fasting plasma glucose level and insulin secretion in non-diabetic males. Moreover, downregulation of *Park2* in rat insulinoma INS-1 β cells caused significant reduction in intracellular ATP level, GSIS, and intracellular insulin expression [90].

Suppressor of cytokine signalling 2 (*SOCS2*) function as modulator of growth factor and cytokine signalling. *SOCS2* is ubiquitously expressed in pancreatic islets [91]. It has been found that the constitutive production of *Socs2* in the β cells of mice causes glucose intolerance and hyperglycaemia by interfering with cytosolic calcium fluxes and proinsulin processing and secretion [92]. Nuclear receptor subfamily 4 group A member 3 (*NR4A3*) plays a role in the regulation of glucose homeostasis. siRNA-mediated knockdown of *NR4A3* in both INS-1 832/13 β cells and human islet was found to prevent cytokine-induced β cell death, whereas overexpression of *NR4A3* caused apoptosis [93]. Phosphotyrosine interacting domain-containing 1 (*PID1*) is a negative regulator of glucose uptake in the muscle and adipose tissue. In addition, under diabetogenic conditions, *Pid1* inactivation has been shown to improve hyperinsulinemia and hyperglycaemia by stimulating glucose uptake in the muscle and adipose tissue of *Pid1*-/- mice [94].

A study was conducted to analyse whole-genome DNA methylation in human pancreatic islets and to identify diabetic islet-specific differentially methylated regions (DMRs). To this end, whole-genome bisulfite sequencing was performed on human islets from patients with T2DM and healthy controls from the Nordic Network for Islet Transplantation. The study identified a total of 25,820 T2DM DMRs, of which 12,124 DMRs exhibited a reduced and 13,696 DMRs exhibited elevated levels of methylation in T2DM islets. The majority of T2DM DMRs were identified in loci with known functions in pancreatic islets, including *ADCY5*, *PDX-1*, and *TCF7L2*. In addition, the study identified 457 genes with DMRs representing significant changes in the gene expression in T2DM islets, including *SLC2A2*, *PARK2*, *SOCS2*, *NR4A3*, and *PID1* [95].

### Blood

#### Dishevelled segment polarity protein 1 and H4 clustered histone 4

Dishevelled segment polarity protein 1 *(DVL1)* has been shown to play a key role in the canonical and non-canonical Wnt signalling pathways and govern several cellular processes including cell survival, proliferation, differentiation, migration, and stem cell renewal [96]. To investigate the role of epigenetic variations in oxidative stress markers and the subsequent development of T2DM and CVD, a genome-wide blood DNA methylation analysis was conducted along with analysis of ten oxidative stress markers. The results identified a total of 66 CpG sites that had an oxidative stress marker-associated DNA methylation profile, with some demonstrating profiles associated with more than one marker. These CpG sites were found to be enriched in the regulatory regions of the genome. The association of these 66 differentially methylated oxidative marker CpG sites with the incident of T2DM during a 10-year follow-up was also investigated. Hypomethylation at the CpG site in the gene body of *DVL1 (*cg03465880) and the CpG site in the 3’-UTR of H4 clustered histone 4 *(HIST1H4D*, cg08170869), was shown to be associated with a higher T2DM risk [97].

#### Thioredoxin interacting protein, ATP-binding cassette sub-family G member 1, and sterile alpha motif domain-containing 12

A family-based study was conducted to investigate the role of epigenome-wide DNA methylation in the pathogenesis of T2DM in blood from members of a high-risk minority population of Mexican-American families. The methylation levels at 51 CpG sites were identified to be significantly associated with T2DM risk, 24 with HOMA-IR and 19 with fasting blood glucose. The top five CpG sites were cg19693031 (3’UTR of *TXNIP*), cg06500161 (*body of ABCG1*), cg07960624 (3’UTR of sterile alpha motif domain-containing 12, *SAMD12*), cg25217710 (intergenic), and cg08309687 (intergenic). These associations remained significant even after adjustments for BMI. Moreover, these five CpG sites were found to account for 7.8% of T2DM heritability in the study cohort [98].

#### ATP-binding cassette sub-family G member 1, lysyl oxidase like 2, thioredoxin-interacting protein, solute carrier family 1 member 5, and sterol regulatory element-binding transcription factor 1

Elevated lysyl oxidase like 2 (*LOXL2*) expression has been associated with tissue fibrosis. As such, ongoing studies are investigating the role of LOXL2 in DM-associated renal fibrosis [99]. A systematic review of recent EWASs linking certain DNA methylation markers to T2DM, HbA1c levels, and fasting blood glucose was conducted. The study replicated the most significant CpG site associations originally identified in the pancreas, liver, peripheral blood, and adipose tissue from the EWASs in blood. The study selected 52 T2DM-associated CpG sites identified in the blood for replication, of which 15 CpG sites demonstrated nominal association with T2DM patients, of which five CpG sites demonstrated a significant association with T2DM after a strict multiple-testing correction. The five CpG sites included cg06500161 (*ABCG1*), cg24531955 (*LOXL2*), cg19693031 (*TXNIP*), cg02711608 (solute carrier family 1 member 5, *SLC1A5*), and cg11024682 (*SREBF1*) [100].

*ABCG1* and *SREBF1* demonstrated hypermethylation, whereas *LOXL2*, *SLC1A5*, and *TXNIP* demonstrated hypomethylation in patients with T2DM compared with healthy controls. However, only the CpG site in *ABCG1* showed a significant association with T2DM after adjustment for BMI. Furthermore, the authors analysed 17 T2DM-associated CpG sites identified in the pancreas and liver in blood samples from the study cohort, and found no significant association for these tissue-specific CpG sites in blood samples, suggesting that DNA methylation patterns in blood may not reflect those of metabolically active tissues [100].

#### Mucosa-associated lymphoid tissue lymphoma translocation protein 1

Mucosa-associated lymphoid tissue lymphoma translocation protein 1 (*MALT1*) is a signalling protein involved in the regulation of insulin and various energy pathways, along with the development and function of B and T cells. This regulation occurs through the MALTI-induced activation of antigen receptor-mediated lymphocytes via the nuclear factor-κB pathway [101]. A genome-wide methylated DNA immunoprecipitation sequencing (MeDIP-seq) study conducted on blood samples identified a number of hypermethylated T2DM-associated DMRs in a discovery group, and replicated the highest signals in a replication group. The strongest signal was annotated to the *MALT1* promoter [101].

#### Fat mass and obesity-associated

The *FTO* gene is believed to play a critical role in the regulation of energy homeostasis, lipolysis, and nucleic acid demethylation. Common genetic variants in *FTO* have been associated with an increased risk of T2DM and obesity [102]. To investigate T2DM-associated DNA methylation, a stepwise study was conducted utilising blood samples. First, a pool-based genome-scale screen identified various previously reported T2DM-associated risk loci to be differentially methylated in T2DM patients. A further in-depth analysis of top-ranking regions was then conducted. The authors found a CpG site (rs1121980) located in the first intron of the *FTO* gene that displayed small but significant hypomethylation in T2DM compared with the controls. In addition, for every 1% decrease in methylation at the CpG site (rs1121980), the odds of belonging to the T2DM group increased by 6.1% [103].

Interestingly, no correlation was observed between *FTO* (rs1121980) DNA methylation and BMI as this locus was hypomethylated in obese and non-obese T2DM patients compared with controls, suggesting that the association between *FTO* (rs1121980) DNA methylation and T2DM may not be mediated through obesity. A follow-up prospective study found a significant hypomethylation of *FTO* (rs1121980) in young individuals that were later diagnosed with T2DM compared with those who remained T2DM-free. This finding not only confirms the initial finding of *FTO* (rs1121980) hypomethylation in patients with T2DM but also suggests that this hypomethylation may represent an early T2DM risk factor rather than a consequence of T2DM. Further genomic analyses demonstrated that *FTO* (rs1121980) colocalises with the binding sites for methylation-sensitive transcriptional regulators and gene enhancers. Taken together, these findings suggest that the T2DM-associated differential methylation of *FTO* (rs1121980) may function as an early T2DM marker. It also suggests that it may function as a distant regulator of gene expression (transcription). However, whether the gene under regulation is *FTO* itself or another (neighbouring or distant) gene remains to be determined [103].

### Adipose tissue

#### Insulin receptor substrate 1, potassium voltage-gated channel subfamily Q member 1, peroxisome proliferator-activated receptor gamma, transcription factor 7-like 2, and others

*IRS1* plays a role in signal transduction from insulin and insulin-like growth factor-1 (IGF) receptors and is involved in glycometabolism. Variants of IRS1 have been shown to be associated with insulin resistance [104]. The *KCNQ1* gene encodes a subunit of a potassium channel that is expressed in human pancreatic β cells, although the role of this channel in insulin secretion is unknown. KCNQ1 has two independent variants, namely, intron 10 and intron 15, that have been associated with an increased risk of T2DM through impaired islet function. However, *KCNQ1* regions are considered T2DM risk factors only when they are maternally inherited. Interestingly, knockdown of KCNQ1 in human islets does not alter insulin secretion [105].

By regulating the expression of several genes, *PPARG* has a number of functions related to the immune response, insulin sensitivity, glucose homeostasis, lipid metabolism, cell fate, and inflammation. In addition, dominant-negative mutations in *PPARG* have been shown to cause severe hyperglycaemia. Furthermore, *PPARG* has been found to be downregulated in T2DM. Studies have also found that PPARG agonists improve insulin sensitivity and glucose tolerance in T2DM patients, reducing the need for β cell insulin secretion and hepatic glucose output and ultimately leading to improved glycaemic control [106].

A genome-wide expression and DNA methylation analysis was conducted on the adipose tissue from monozygotic twin pairs discordant for T2DM and independent case–control cohorts. In the adipose tissue from the monozygotic twin pairs, the authors found an increased expression of the genes involved in glycan degradation and inflammation and decreased expression of the genes involved in carbohydrate, lipid, amino acid metabolism, and those involved in oxidative phosphorylation in diabetic verses non-diabetic co-twins. The most differentially expressed genes included C-C motif chemokine ligand 18 (*CCL18*), ELOVL fatty acid elongase 6 (*ELOVL6*), fatty acid desaturase 1 (*FADS1*), glycogen synthase 2 (*GYS2*), interleukin 1 receptor antagonist (*IL1RN*), and secreted phosphoprotein 1 (*SPP1*). These results were successfully replicated in the adipose tissue from an independent case–control cohort of unrelated subjects with T2DM or NGT. Moreover, a number of candidate genes previously linked through GWAS to T2DM and obesity were found to be differentially expressed in the adipose tissue from discordant twins, including *PPARG*, GLIS family zinc finger 3 (GLIS3) (T2DM), vascular endothelial growth factor A (*VEGFA*), and *IRS1* (obesity) [107].

In addition, the study identified 15,627 differentially methylated CpG sites in the adipose tissue from unrelated subjects with T2DM compared with control subjects. These sites represented 7046 genes, including *IRS1*, *KCNQ1*, *PPARG*, and *TCF7L2*. Interestingly, 6754 of the 15,627 sites showed increased methylation, whereas the remaining 8873 sites showed decreased methylation in patients with T2DM. In addition, 1410 of the 15,627 sites showed differential DNA methylation levels in the twins discordant for T2DM, and approximately 50% of the genes differentially expressed in the discordant twins were associated with DNA methylation [107].

However, the findings of this study investigating the differential expression and methylation of *IRS1* in T2DM contradicted those from a previous single-gene study, in which the association between the DNA methylation of three CpG dinucleotides within the *IRS1* promoter and increased risk of T2DM was investigated. In this single-gene study, blood samples from patients with T2DM and healthy (control) individuals were used. Surprisingly, no significant association between *IRS1* promoter methylation and increased risk of T2DM was observed. A breakdown analysis by gender also revealed no association with T2DM, although two CpG dinucleotides showed significantly lower methylation levels in males compared with females [104].

### Multiple tissues/organs

#### Musashi RNA binding protein 2

The Mushashi (MSI) protein is an RNA-binding protein that has two isoforms, namely, MSI1 and MSI2, and exerts a regulatory effect on the transcription of the insulin gene and β cell proliferation [108].

A study was conducted to investigate the relationship between glucose homeostasis and differential DNA methylation using the OGTT in a population-based cohort, where all the subjects were recruited from the Korean Genome Epidemiology Study (KoGES). In this study, the subjects were divided into two subgroups, namely, a hyperglycaemic group and a T2DM group, which were then compared with a control group. Blood samples were taken for analysis, and non-tumour human pancreatic islet samples were obtained from pancreatectomy procedures performed at the Asian Medical Centre in Seoul, Korea. The results of the study found a total of 382 differentially methylated positions in the blood samples from the two subgroups, annotating to 280 genes. Among them, three CpG sites were overlapped in the two subgroups. Two of these sites were found to be hypomethylated and were mapped to the *MSI2* and CXXC finger protein-4 (*CXXC4)* genes. A further analysis showed that *MSI2* was significantly associated with T2DM, with the DNA methylation of this gene considerably decreased in the blood and pancreatic islets from the T2DM donors. In addition, increased *MSI2* DNA methylation was positively correlated with increased insulin sensitivity (*QUICKI*) and decreased insulin resistance (HOMA-IR) [109].

This differentially hypomethylated CpG site (chr17:55484635) is located within intron 6 of the *MSI2* gene. The DNA methylation analysis of the 280 CpG sites in the *MSI2* promoter found no significant differences in terms of methylation, and none of the sites were found to be associated with T2DM. From these findings, it was hypothesised that the differential methylation at chr17:55484635 may not be directly related to *MSI2* expression. However, no gene expression analysis was conducted in this study to confirm or deny this conclusion. The authors also suggested that the hypomethylation at chr17:55484635 may affect the expression of other genes [109].

A previous study [108] characterised the presence and function of the two Musashi isoforms in pancreatic islets and β cells. Although this study did not address the methylation status of the *MSI2* gene, it did find the expression of *Msi2* in mouse insulinoma Min6 β cells to be unaffected by either hypoglycaemic or hyperglycaemic conditions. In these cells, *MSI2* overexpression significantly downregulated *Ins1* and *Ins2* gene expressions and blocked MIN6 proliferation, while *Msi2* knockdown resulted in the upregulation of *Ins2* expression and augmented MIN6 proliferation [108].

#### Nuclear receptor subfamily 4 group A, member 1

Nuclear receptor subfamily 4 group A, member 1 (*NR4A1*) is a transcriptional regulator for glucose metabolism present in the skeletal muscle and liver [110].

The evaluation of the role of *DNMT1* in T2DM was performed as part of a genome-wide DNA methylation array study, utilising blood samples from patients with T2DM and healthy (control) individuals, in addition to RIN-m5F rat pancreatic β cells, 293T cells, and male KK and KK.Cg-*A*^*y*^/J mice (as a T2DM animal model). This study found that the promoter of the *NR4A1* gene was hypermethylated in the patients with T2DM and resulting in a reduced *NR4A1* mRNA expression. *Nr4a1* was also found to be hypermethylated in a mouse T2DM model [110].

In addition, the transient transfection of human *NR4A1* into the RIN-m5F and 293T cells was shown to induce the inhibition of *DNMT1* and the overexpression of the insulin receptor. The short hairpin RNA (shRNA)-mediated knockdown of *NR4A1 in* RIN-m5F and 293T cells resulted in *DNMT1* induction and insulin receptor downregulation in RIN-m5F cells, suggesting that *NR4A1* gene is somehow part of the overall epigenetic regulation of the insulin-signalling pathway [110].

The T2DM mouse model showed a reduced activation of pancreatic β cell Dnmt1 and reduced *Nr4a1* hypermethylation when treated with the DNMT1 inhibitor aurintricarboxylic acid (ATA). This reduction managed to reverse the changes in *Nr4a1* expression and reduce blood glucose levels in the mice. Therefore, the authors of the study hypothesised that these findings may represent a possible novel therapy, through the utilisation of ATA to reverse the impaired NR4A1-dependent insulin signalling and lower the blood glucose levels in patients with T2DM [110].

## Other regulatory elements

T2DM-specific DNA methylation profiles are not limited to genes, but rather extend to some non-gene genomic sequences. For example, *Alu* elements DNA methylation was found to be significantly reduced in blood from T2DM, with *Alu* DNA hypomethylation directly correlating with high HbA1C and high fasting blood glucose [111]. In addition, lower blood DNA methylation levels of long interspersed nucleotide element 1 (LINE-1) were associated with an increased metabolic risk, independent of the classic risk factors (e.g., age, sex, physical activity, and BMI) [112]. However, a more recent study reported opposite findings, where higher blood DNA methylation levels of LINE-1 were found in T2DM patients compared to control, and lower blood DNA methylation levels of LINE-1 were associated with reduced T2DM risk [113].

Finally, DNA hypermethylation of the micro RNA (miRNA) cluster in the imprinted 14q32 locus was found to substantially downregulate its expression in T2DM pancreatic β cells. The expression of this miRNA cluster was found to play an important role in regulating the expression of two T2DM-relevant genes, namely tumour protein p53-inducible nuclear protein-1 (*TP53INP1*) and islet amyloid polypeptide (*IAPP*). These findings indicate an important role for miRNAs in the pathogenesis of T2DM, along with their epigenetic regulation by DNA methylation [114].

## Conclusion

In this review, we provide examples of a long list of genes whose altered DNA methylation profiles have been associated with the pathogenesis of T2DM (Table 1). In addition, we discuss the T2DM-related altered DNA methylation profiles associated with these genes. However, altered epigenetic profiles include alterations in other epigenetic modifications, along with the aberrant expression of non-coding RNAs and miRNAs.

**Table 1:** List of T2DM-associated genes and their methylation status

Gene	Species	Organ/tissue	Sample size	Findings	Reference
*Ins*	Human	Pancreatic islets	Non-diabetic = 48, T2DM = 9	Hypermethylation	[18]
	Rat	INS 832/13 β cell line			
	Rat	INS-1 β cell line		Hypermethylation	[12]
	Rat	Pancreatic islets from Zucker diabetic fatty rats	10 rats		
	Human	Pancreatic islets	Non-diabetic = 34, T2DM = 15	Hypermethylation	[86]
*PDX-1*	Human	Pancreatic islets	Non- diabetic = 55, T2DM = 9	Hypermethylation	[24]
	Rat	INS 832/13 β cell line			
	Human	Pancreatic islets	Non-diabetic = 34, T2DM = 15	Hypermethylation	[86]
*PPARGC1A*	Human	Pancreatic islets	Non-diabetic = 48, T2DM = 12	Hypermethylation	[30]
*FOSL2*	Human	Blood	NGT = 50, T2DM = 50	Hypermethylation	[31]
*PTPN1*	Human	Blood	Non-diabetic = 97, T2DM = 97	Hypermethylation	[33]
*SLC30A8*	Human	Blood	NGT = 27, T2DM = 161	Hypermethylation	[40]
*IGF-1*	Human	Blood	NGT = 242, T2DM = 164	Hypermethylation	[42]
*IGFBP-1*	Human	Blood	NGT = 242, T2DM = 164	Hypermethylation	[44]
*IGFBP-7*	Human	Blood	NGT = 100, T2DM =240	Hypermethylation	[46]
*GCK*	Human	Blood	Non-diabetic = 47, T2DM = 47	Hypermethylation	[52]
*MCP-1*	Human	Blood	Non-diabetic = 15, T2DM = 32	Hypomethylation	[55]
*TP53*	Human	Blood	Non-diabetic = 12, T2DM = 27	Hypermethylation	[56]
*BRCA1*	Human	Blood	Non-diabetic = 12, T2DM = 27	Hypermethylation	[56]
*SCARA3*	Human	Blood	Non-diabetic = 12, T2DM = 27	Hypermethylation	[56]
*PRDX2*	Human	Blood	Non-diabetic = 12, T2DM = 27	Hypermethylation	[56]
*PRKCZ*	Human	Blood	Non-diabetic = 120, T2DM = 152	Hypermethylation	[58]
*GIPR*	Human	Blood	Non-diabetic = 93, T2DM = 93	Hypomethylation	[60]
*PTPRD*	Human	Blood	Non-diabetic = 98, T2DM = 94	Hypermethylation	[70]
	Mouse	Liver tissue from KK and KK-Cg-*A*^*y*^/J mice			
*PDK4*	Human	Blood	Normal = 11, T2DM = 25, MetS = 9	Hypomethylation (in T2DM+MetS groups vs control)	[73]
	Human	Skeletal muscle	NG = 79, T2DM = 33	Hypomethylation	[74]
*PPARG*	Human	Blood	Normal = 11, T2DM = 25, MetS = 9	Hypermethylation	[73]
*ABCG1*	Human	Blood	19 monozygotic twins discordant for T2DM	Hypermethylation	[77]
	Human	Adipose tissue	14 monozygotic twins discordant for T2DM		
	Human	Blood	Non-diabetic = 98, T2DM = 100	Hypermethylation	[100]
*PHOSPHO1*	Human	Skeletal muscle	17 monozygotic twins discordant for T2DM	Hypomethylation	[77]
*TXNIP*	Human	Blood	19 monozygotic twins discordant for T2DM	Hypomethylation	[77]
	Human	Skeletal muscle	17 monozygotic twins discordant for T2DM		
	Human	Pancreatic islets	Non-diabetic = 34, T2DM = 15		
	Human	Blood	**Discovery:** BASICMAR_1 cohort: non-DM = 204, DM = 151 **Replication:** BASICMAR_2 cohort: non-DM = 108, DM = 59 REGICOR cohort: non-DM = 582, DM = 63	Hypomethylation	[82]
	Human	Blood	Non-diabetic = 98, T2DM = 100	Hypomethylation	[100]
*SREBF1*	Human	Pancreatic islets	Non-diabetic = 34, T2DM = 15	Hypermethylation	[77]
	Human	Blood	Non-diabetic = 98, T2DM = 100	Hypermethylation	[100]
*EXOC3L2*	Human	Pancreatic islets	Non-diabetic = 34, T2DM = 15	Hypermethylation	[86]
*CDKN1A*	Human	Pancreatic islets	Non-diabetic = 34, T2DM = 15	Hypomethylation	[86]
*PDE7B*	Human	Pancreatic islets	Non-diabetic = 34, T2DM = 15	Hypomethylation	[86]
*SEPT9*	Human	Pancreatic islets	Non-diabetic = 34, T2DM = 15	Hypomethylation	[86]
*SLC2A2*	Human	Pancreatic islets	Non-diabetic = 8, T2DM = 6	Hypermethylation	[95]
*DVL1*	Human	Blood	966 individuals from the general population	Hypomethylation	[97]
*HIST1H4D*	Human	Blood	966 individuals from the general population	Hypomethylation	[97]
*LOXL2*	Human	Blood	Non-diabetic = 98, T2DM = 100	Hypomethylation	[100]
*SLC1A5*	Human	Blood	Non-diabetic = 98, T2DM = 100	Hypomethylation	[100]
*FTO*	Human	Blood	Pool-based cross-sectional study: Non-diabetic = 459, T2DM = 710 Individual-based cross-sectional study: Non-diabetic = 233, T2DM = 198 Longitudinal study: 515 individuals	Hypomethylation	[103]
*MSI2*	Human	Blood	Discovery group: Non-diabetic = 13, hyperglycaemic = 8, T2DM = 5 Replication group: Non-diabetic = 220, T2DM = 220	Hypomethylation	[109]
	Human	Pancreatic islets	Non-diabetic = 16, T2DM = 2		
*NR4A1*	Human	Blood	Non-diabetic = 98, T2DM = 94	Hypermethylation	[110]
	Mouse	Pancreatic islets and blood from KK and KK.Cg-*A*^*y*^/J mice			

In conclusion, although T2DM is a considerably complex disease, the above-mentioned studies provide some insights into the significant role played by epigenetics and DNA methylation in particular in the pathogenesis of T2DM, including insulin production, β cell secretion, and resistance. This regulation can either occur by affecting the DNA methylation of the insulin gene itself or by affecting the DNA methylation status of other genes that regulate insulin production, secretion, and sensing. Accordingly, these recent findings highlight a promising new route in terms of the development of novel therapeutics for T2DM, targeting DNA methylation in a way that enhances pancreatic β cell insulin production and secretion, along with targeting insulin sensitivity.

## Future scope

Disease-associated epigenetic profiles can be considered either causative of the disease or the consequence (or a combination of both). Similar to the chicken and egg paradox, dissecting whether the disease or epigenetic profile came first is often hard. Accordingly, Volkmar et al. [115], pointed out that the methylation landscape, although associated with T2DM, can be either one of the contributing factors in the development of T2DM or the result of the reaction of β cells to T2DM hyperglycaemia.

In this review, we discussed the T2DM-associated DNA methylation profiles, but these profiles only represent a fraction of the bigger picture. Indeed, the overall T2DM-associated altered epigenetic profile includes alterations in several, if not all, of the epigenetic modifications identified to date. Accordingly, a number of altered histone tail modification profiles, along with those of altered non-coding RNAs, such as miRNAs, have been associated with the pathogenesis of T2DM. In addition to T2DM, a number of diseases have been found to be associated with altered epigenetic profiles. Furthermore, different types of epigenetic modifications likely do not operate in isolation but rather work together as they crosstalk with and regulate each other. Hence, a revisit to the already identified genes to analyse the entire epigenetic profile of each gene, along with the expressions of their respective regulators, may prove beneficial.

In addition, the majority of the DNA profiles discussed in this review were identified using peripheral blood samples. However, some DNA methylation profiles (and even entire epigenetic profiles) are tissue specific. Thus, further in-depth analysis of DNA methylation profiles in T2DM-specific tissues, such as the pancreas, liver, and skeletal muscle, is warranted.

Taken together, the identified changes in the DNA methylation profiles (or the entire epigenetic profiles) of certain genes may be further utilised to develop T2DM risk assessments and/or prognosis prediction tools in either a generic, population, or race-specific manner. Such novel findings, like the ones discussed in this review, are carving a new route for the development of novel therapeutics for T2DM targeting DNA methylation in a way that enhances pancreatic β cell insulin production and secretion, along with targeting insulin sensitivity. A greater understanding of the role of epigenetics in T2DM is expected to lead to improved interventions and treatments, which will hopefully reduce the global incidence of T2DM.

## Data Availability

Not applicable

## Abbreviations

5’-CpG-3’: 5’-cytosine-phosphodiester bond-guanine-3’
5-Aza-CdR: 5-aza-2’-deoxycytidine
5mC: 5-methylcytosine
ABCG1: ATP-binding cassette subfamily G member 1
ADAMTS9: ADAM metallopeptidase with thrombospondin type 1 motif 9
ADCY5: Adenylate cyclase 5
AID: Activation induced deaminase
Arx: Aristaless-related homeobox
ATA: Aurintricarboxylic acid
ATF2: Activating transcription factor 2
AVPR2: *A*rginine vasopressin receptor 2
BCL11A: B cell CLL/lymphoma 11A
BER: Base excision repair
BMI: Body mass index
bp: Base pairs
BRCA1: Breast cancer 1
CALM2: Calmodulin 2
cAMP: Cyclic adenosine monophosphate
CCL18: C-C motif chemokine ligand 18
CDC123/CAMK1D: Cell division cycle protein 123/calcium/calmodulin-dependent protein kinase 1D
CDKAL1: CDK5 regulatory subunit-associated protein 1-like 1
CDKN1A: Cyclin-dependent kinase inhibitor 1A
CDKN2A: Cyclin-dependent kinase inhibitor 2A
-CH_3_: Methyl group
CHCHD9: Coiled-coil-helix-coiled-coil-helix domain-containing 2 pseudogene 9
CRE: cAMP responsive element
CREB-1: cAMP responsive element-binding protein-1
CRY2: Cryptochrome 2
CVD: Cardiovascular disease
CXXC4: CXXC finger protein-4
DM: Diabetes mellitus
DM-MetS: DM plus MetS
DMRs: Differentially methylated regions
DN: Diabetic nephropathy
DNMT: DNA methyltransferase
DUSP8: Dual specificity phosphatase 8
DUSP9: Dual specificity phosphatase 9
DVL1: Dishevelled segment polarity protein 1
ELOVL6: ELOVL fatty acid elongase 6
EWAS: Epigenome-wide association study
EXOC3L2: Exocyst complex component 3-like 2
FADS1: Fatty acid desaturase 1
FOSL2: FOS-like antigen 2
FTO: Fat mass and obesity-associated
GCK: Glucokinase
GDM: Gestational DM
GIP: Gastric inhibitory polypeptide
GIPR: Gastric inhibitory polypeptide receptor
GLIS3: GLIS family zinc finger 3
GLP-1R: GLP-1 receptor
GRB10: Growth factor receptor-bound protein-10
GSIS: Glucose-stimulated insulin secretion
GWAS: Genome-wide association studies
GYS2: Glycogen synthase 2
HbA1c: Glycated haemoglobin A1c
HHEX: Haematopoietically expressed homeobox
HIST1H4D: Histone cluster 1, H4d
HMGA2: High-mobility group AT-hook 2
HOMA-B: Homeostatis model assessment of β-cell function
HOMA-IR: Homeostasis model assessment of insulin resistance
IAPP: Islet amyloid polypeptide
IGF-1: Insulin-like growth factor-1
IGFBP-1: Insulin-like growth factor-binding protein 1
IGFBP-7: Insulin-like growth factor-binding protein 7
IL1RN: Interleukin 1 receptor antagonist
Ins: Insulin
IRS1: Insulin receptor substrate 1
Kb: Kilobase pairs
KCNJ11: Potassium voltage-gated channel subfamily J member-11
KCNQ1: Potassium voltage-gated channel subfamily Q member-1
LINE-1: Long interspersed nucleotide element 1
LOXL2: Lysyl oxidase like 2
MALT1: Mucosa-associated lymphoid tissue lymphoma translocation protein 1
MBD2: Methyl-CpG-binding domain protein 2
MCP-1: Monocyte chemoattractant protein-1
MeCP2: Methyl CpG binding protein 2
MeDIP-seq: Methylated DNA immunoprecipitation sequencing
MetS: Metabolic syndrome
miRNA: MicroRNA
MSI2: Musashi RNA binding protein 2
MTNR1B: Melatonin receptor 1B
NGT: Non-impaired (normal) glucose tolerance
NR4A1: Nuclear receptor subfamily 4 group A, member 1
NR4A3: Nuclear receptor subfamily 4 group A member 3
OGTT: Oral glucose tolerance test
PDC: Pyruvate dehydrogenase complex
PDE7B: Phosphodiesterase-7B
PDK4: Pyruvate dehydrogenase kinase
PDX-1: Pancreatic and duodenal homeobox-1
PEG3: Paternally expressed gene-3
PHOSPHO1: *Phosphoethanolamine/phosphocholine phosphatase 1*
PID1: Phosphotyrosine interacting domain-containing 1
PPARG / PPARγ: Peroxisome proliferator-activated receptor gamma
PPARGC1A: Peroxisome proliferator-activated receptor gamma coactivator-1 alpha
Prdx2: *P*eroxiredoxin 2
PRKCZ: Protein kinase C epsilon zeta
PARK2: Parkin RBR E3 ubiquitin protein ligase
PTPN1: *P*rotein tyrosine phosphatases non-receptor type 1
PTPRD: Protein tyrosine phosphatases receptor type D
RPA1: Replication protein A1
SAM: S-adenosyl methionine
SAMD12: Sterile alpha motif domain-containing 12
SCARA3: Scavenger receptor class A member 3
SCD1: Stearoyl-coenzyme A desaturase-1
SEPT9: Septin-9
shRNA: Short hairpin RNA
siRNA: Small interfering RNA
SLC2A2: Solute carrier family 2 member 2
SLC30A8: Solute carrier family 30 member 8
SOCS2: Suppressor of cytokine signalling 2
SOCS3: Suppressor of cytokine signalling 3
SPP1: Secreted phosphoprotein 1
SREBF1: Sterol regulatory element-binding transcription factor 1
SRR: Serine racemase
T1DM: Type 1 DM
T2DM: Type 2 DM
TCF7L2: Transcription factor 7-like 2
TETs: Ten-eleven translocations
THADA: THADA armadillo repeat containing
Tp53: Tumour protein p53
TP53INP1: Tumour protein p53-inducible nuclear protein-1
TSPAN8: Tetraspanin 8
TSS: Transcription start site
TXNIP: Thioredoxin-interacting protein
VEGFA: Vascular endothelial growth factor A
WFS1: Wolframin ER transmembrane glycoprotein
ZBED3: *Z*inc finger BED-type containing 3
ZnT-8: Zinc transporter 8
